# Lower risk of dementia with AS01-adjuvanted vaccination against shingles and respiratory syncytial virus infections

**DOI:** 10.1038/s41541-025-01172-3

**Published:** 2025-06-25

**Authors:** Maxime Taquet, John A. Todd, Paul J. Harrison

**Affiliations:** 1 Department of Psychiatry, University of Oxford, Oxford, UK; 2 Oxford Health NHS Foundation Trust, Warneford Hospital, Oxford, UK; 3 Centre for Human Genetics, Nuffield Department of Medicine, University of Oxford, Oxford, UK

**Keywords:** Dementia, Adjuvants

## Abstract

AS01-adjuvanted shingles (herpes zoster) vaccination is associated with a lower risk of dementia, but the underlying mechanisms are unclear. In propensity-score matched cohort studies with 436,788 individuals, both the AS01-adjuvanted shingles and respiratory syncytial virus (RSV) vaccines, individually or combined, were associated with reduced 18-month risk of dementia. No difference was observed between the two AS01-adjuvanted vaccines, suggesting that the AS01 adjuvant itself plays a direct role in lowering dementia risk.

There is growing evidence that vaccination against shingles (also known as herpes zoster, which is caused by reactivation of the varicella-zoster virus) protects against dementia^1–7^, with some studies showing a stronger effect in females^5,7^. Our recent natural experiment of over 100,000 people in the US Electronic Health Record (EHR) database, TriNetX, found that the Adjuvant System AS01 shingles vaccine (also referred to as the recombinant shingles vaccine, or Shingrix)^8^ was associated with a lower risk of dementia than the live vaccine (Zostavax; now discontinued in the USA and many other countries because of greater efficacy of Shingrix) in both males and females^5^. Two non-mutually exclusive hypotheses can explain the added protection provided by the adjuvanted vaccine compared to the live vaccine (which has no adjuvant): shingles might increase the risk of dementia^9^, and the adjuvanted vaccine would therefore better protect against dementia through its greater efficacy; and/or the AS01 adjuvant might itself provide some protection against dementia as suggested by mouse models^10,11^. Understanding the relative contributions of these possibilities would be important for translating findings into potential strategies for the prevention of Alzheimer’s disease and other neurodegenerative disorders.

Here, we analysed EHR data to test the possibility that AS01 contributes to the reduction in risk of dementia seen with Shingrix by also investigating the effect of the AS01-containing RSV vaccine (Arexvy). The two vaccines were compared against each other and against the flu vaccine, in terms of the risk of a dementia diagnosis in the following 18 months.

A total of 35,938 who received the AS01 RSV vaccine only (mean [SD] age: 72.8 [7.0] years, 58.1% female), 103,798 people who received the AS01 shingles vaccine only (mean [SD] age: 69.2 [7.0] years, 54.9% female) and 78,658 who received both (mean [SD] age: 72.4 [7.0], 57.8% female) were adequately matched (all standardised mean differences [SMD] < 0.1) to an equal number of people who received the flu vaccine and neither shingles nor RSV vaccine (Table 1 and Supplementary Data 1).

**Table 1:** Baseline characteristics for the different cohorts after matching

	RSV vaccine vs flu vaccine		Shingles vaccine vs flu vaccine		RSV + shingles vaccine vs flu vaccine	
	RSV vaccine	Flu vaccine	Shingles vaccine	Flu vaccine	RSV + shingles vaccine	Flu vaccine
**Number**	35,938	35,938	103,798	103,798	78,658	78,658
**Sociodemographics**						
Age; mean (SD); y	72.84 (7.05)	72.91 (7.06)	69.20 (7.03)	69.25 (6.99)	72.44 (6.96)	72.42 (6.98)
Sex; *n* (%)						
Female	20,883 (58.11)	20,878 (58.09)	56,975 (54.89)	57,051 (54.96)	45,459 (57.79)	45,410 (57.73)
Male	15,008 (41.76)	15,023 (41.80)	46,736 (45.03)	46,664 (44.96)	33,166 (42.16)	33,214 (42.23)
Race; *n* (%)						
White	27,897 (77.62)	27,971 (77.83)	75,412 (72.65)	75,303 (72.55)	63,230 (80.39)	62,885 (79.95)
Black or African American	3858 (10.73)	3771 (10.49)	13,350 (12.86)	13,362 (12.87)	5396 (6.86)	5565 (7.08)
Asian	1782 (4.96)	1754 (4.88)	5688 (5.48)	5507 (5.30)	4761 (6.05)	4605 (5.85)
Unknown	1351 (3.76)	1361 (3.79)	4656 (4.49)	4906 (4.73)	2434 (3.09)	2797 (3.56)
**Comorbidities [ICD-10 code];** ***n*** **(%)**						
Haematological conditions [D50–89]	14,889 (41.43)	14,609 (40.65)	36,880 (35.53)	36,590 (35.25)	30,657 (38.98)	29,803 (37.89)
Thyroid disorders [E00-07]	10,319 (28.71)	10,025 (27.89)	25,563 (24.63)	25,247 (24.32)	21,376 (27.18)	20,634 (26.23)
Diabetes mellitus [E08-13]	10,791 (30.03)	10,482 (29.17)	29,282 (28.21)	29,041 (27.98)	21,600 (27.46)	20,906 (26.58)
Vitamin B deficiency [E53]	4062 (11.30)	3974 (11.06)	8751 (8.43)	8744 (8.42)	7332 (9.32)	6987 (8.88)
Vitamin D deficiency [E55]	12,210 (33.98)	11,789 (32.80)	30,226 (29.12)	30,238 (29.13)	22,039 (28.02)	21,411 (27.22)
Overweight and obesity [E66]	12,192 (33.92)	11,895 (33.10)	35,866 (34.55)	35,576 (34.27)	26,507 (33.70)	25,755 (32.74)
Metabolic disorders [E70–88]	27,589 (76.77)	26,993 (75.11)	79,188 (76.29)	78,565 (75.69)	57,486 (73.08)	55,665 (70.77)
Substance use disorder [F10–19]	6005 (16.71)	5896 (16.41)	19,948 (19.22)	19,636 (18.92)	11,961 (15.21)	11,837 (15.05)
Mood disorder [F30-39]	8826 (24.56)	8665 (24.11)	26,709 (25.73)	26,304 (25.34)	19,726 (25.08)	19,153 (24.35)
Hypertension [I10]	22,206 (61.79)	21,680 (60.33)	64,288 (61.94)	63,881 (61.54)	46,719 (59.40)	44,971 (57.17)
Ischaemic heart disease [I20–25]	7179 (19.98)	6978 (19.42)	21,571 (20.78)	21,343 (20.56)	18,218 (23.16)	17,600 (22.38)
Cerebrovascular diseases [I60–69]	5292 (14.72)	5225 (14.54)	12,235 (11.79)	11,987 (11.55)	10,030 (12.75)	9699 (12.33)
Respiratory disease [J00–99]	23,166 (64.46)	22,734 (63.26)	62,588 (60.30)	61,951 (59.68)	47,529 (60.42)	45,921 (58.38)
Falls [W0–19]	5641 (15.70)	5482 (15.25)	12,731 (12.27)	12,502 (12.04)	11,655 (14.82)	11,336 (14.41)

Only a subset of representative characteristics is presented. All baseline characteristics are displayed in Supplementary Data 1. All SMD were <0.1.

Compared to those who received the flu vaccine, those who received the RSV or shingles vaccines were at a lower risk of being diagnosed with dementia (ratio of RMTL: 0.71, 95% confidence interval [CI] 0.61–0.83, *P* = 2.8 × 10^−5^ for RSV only; 0.82, 95% CI 0.74–0.91, *P* = 0.00027 for shingles only; and 0.63, 95% CI 0.55–0.72, *P* = 4.7 × 10^−12^ for those who received both vaccines; Figs. 1 and 2). These correspond to 29%, 18% and 37% additional time spent diagnosis-free, or 87, 53 and 113 days for those diagnosed within 18 months of vaccination, respectively. There was no significant difference in the risk of dementia between those who received either AS01 vaccine and those who received both (Figs. 1A and 2). The risk of a composite negative control outcome did not differ between cohorts, and the risk of shingles was lower in those who received the shingles vaccine (Fig. 1A). Results were similar in males and females (with no moderation of the effects by sex: *P* = 0.40 for shingles vaccination only, *P* = 0.46 for RSV vaccination only, and *P* = 0.14 for the combination of shingles and RSV vaccination), and when including those who developed dementia during the first 3 months of follow-up (Fig. 1B). Results were similar for the composite outcome of dementia or death (Supplementary Data 2). Results in terms of dementia subcategories had wide CIs, reflecting the low incidence of some subcategories and the lack of use of subcodes in routine clinical practice, except for ‘Unspecified dementia’ that mirrored the primary finding (Supplementary Data 2).

**Fig. 1:**
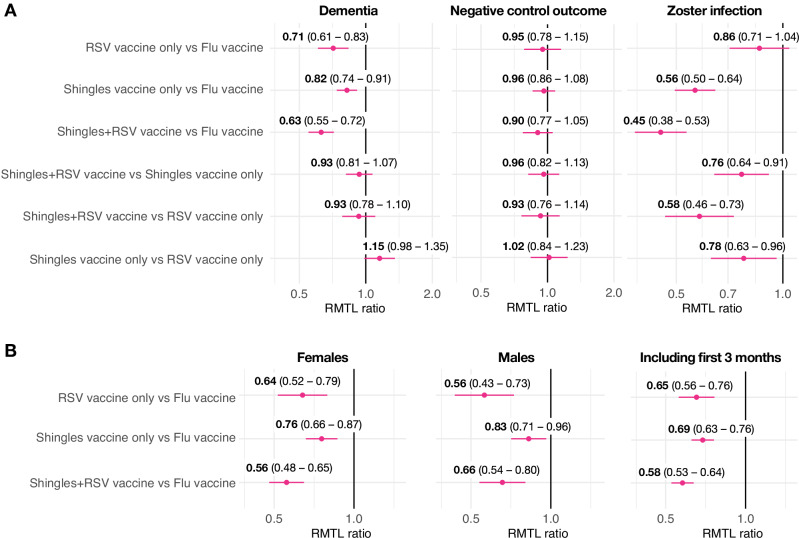
Associations between AS01 vaccines and dementia, negative control outcome and zoster infection in the primary and secondary analyses. **A** Association between AS01-adjuvanted vaccines and risk of dementia, negative control outcome and zoster infection. Each dot and bold number represent the ratio of restricted mean time lost (RMTL) for the comparison between two cohorts, while horizontal lines and numbers in brackets are 95% confidence intervals. RMTL ratios below 1 indicate that the risk is lower in the first cohort (e.g. recipients of the RSV vaccine on the first line) than in the second (e.g. recipients of the flu vaccine). **B** Associations between AS01-adjuvanted vaccines (compared to flu vaccine) and risk of dementia in females, males and in the cohorts including people who developed dementia within the first 3 months post-vaccination.

**Fig. 2:**
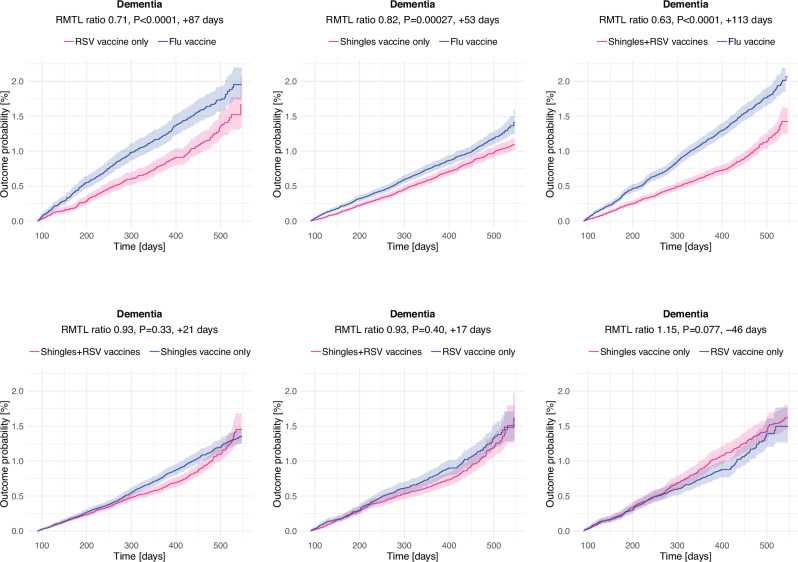
Kaplan–Meier estimates of the cumulative incidence of dementia in the comparison between cohorts. Shaded areas represent 95% CI. The RMTL ratio, the *P* value for the association, and the additional time that affected people lived diagnosis-free are reported above each figure.

The mechanisms underlying the protective effects of both AS01 vaccines against dementia remain unclear. One possibility is that they independently reduce dementia risk by preventing their respective infections, shingles and RSV. Growing evidence suggests that infections, including RSV infections^12^ and shingles^9^ (both of which can be neurotropic^13,14^) may increase dementia risk, so that vaccinating against them could reduce this risk. However, the fact that a protective effect is seen within a few months of vaccination (as was observed in our previous study^5^) argues against this possibility as it seems unlikely that enough infections would be prevented during such a short time frame to explain the magnitude of the protection against dementia. In addition, if the adjuvanted vaccines exerted their protective effect via separate mechanisms, we would expect an additive protective effect in individuals who received both vaccines compared to those who received only one—a pattern not observed here. Furthermore, the mixed evidence for a causal link between shingles and dementia^15–18^ suggests that the AS01 shingles and RSV vaccines protect against dementia through mechanisms unrelated to (or at least in addition to) the prevention of their viral antigen-specific target infection. As such, the adjuvant they share is a plausible explanation.

AS01 might protect against dementia via specific immunological pathways. In particular, toll-like receptor 4 stimulation with monophosphoryl lipid A (MPL; one of the components of the AS01 system) has been shown to improve Alzheimer’s disease pathology in mice^11^. In addition, the two main ingredients of AS01, MPL and QS-21 (a purified plant extract derived from Quillaja saponaria), act synergistically to activate macrophages and dendritic cells^19^ and trigger an age-independent cytokine cascade that culminates in the production of interferon gamma (IFN-γ)^19,20^. IFN-γ might attenuate amyloid plaque deposition (as seen in mice^21^) and is negatively correlated with cognitive decline in cognitively unimpaired older adults^22^. It might be that these neuroprotective mechanisms reach their full potential at or below the dose of AS01 administered within a single vaccine, so that administering both the AS01 shingles and RSV vaccines does not provide further benefits. This saturation effect could also explain why the level of protection against dementia appears similar between the AS01 shingles vaccine (that is given in two doses) and the AS01 RSV vaccine (administered as a single dose).

This study has several strengths, including large sample sizes, matching for a range of confounding factors, and the inclusion of different exposures to triangulate the evidence. It also has several limitations in addition to those inherent to studies based on EHR data, such as no validation of diagnoses and sparse information on socioeconomic and lifestyle factors. First, being diagnosis-free does not mean being disease-free, because there can be diagnostic delays. However, if diagnostic delays are similar between cohorts, then differences in disease-free time will follow differences in diagnosis-free time. Second, we did not investigate the impact of several doses of the vaccine. Third, the code used to identify the RSV vaccine in the EHR dataset covers both the RSVPreF3 OA vaccine *Arexvy* (AS01 RSV vaccine) and a smaller proportion of the RSVpreF vaccine *Abrysvo* (a recombinant RSV vaccine not containing AS01). The brand is only specified for 44% of occurrences, and so our cohort likely includes people who received Abrysvo (estimated to account for 24% of the whole RSV vaccinated cohort). This suggests that we are underestimating the actual protective effect of *Arexvy*. Fourth, while this study aims to estimate potential causal effects, its reliance on observational data means the findings may be affected by bias due to unmeasured confounding. The null associations with a negative control outcome and the observation of expected associations with zoster infection support the absence of obvious bias but cannot rule it out.

In summary, taking the current results and other reports into account, we conclude that it is likely that both the AS01 shingles and RSV vaccines provide some protection against dementia. The mechanisms underpinning this protection remain to be determined. Our data provide support for the hypothesis that, besides protection against their target infection, these vaccines could well protect against dementia via the action of the AS01 components through specific immunological pathways. These findings justify further clinical and mechanistic studies to confirm and understand the protective effects and their duration.

## Methods

### Study design and data source

We conducted retrospective cohort studies using TriNetX US Collaborative Network, a federated EHR network with anonymised data from 120 million patients in 69 healthcare organisations in the USA. The participating healthcare organisations are a mixture of hospitals, primary care and specialist providers, including insured and uninsured patients, and both academic and non-academic centres. This implies that the sample is at least broadly representative of the American population, but generalisation beyond the USA requires replication in other cohorts. The process of data de-identification is attested by a qualified expert as defined in Section 164.514(b) of the Health Insurance Portability and Accountability Act of 1996 (HIPAA) Privacy Rule. No further ethical approval was needed. As we used anonymised, routinely collected data, no participant consent was required.

### Cohorts and exposures

Three primary cohorts were defined: individuals who received only the AS01 RSV vaccine, those who received only the AS01 shingles vaccine, and those who received both vaccines (with the RSV vaccine following the shingles vaccine by a median time of 4 years). The index date for follow-up was the vaccination date (set as the date of the RSV vaccine for those who received both). The index date had to be on or after 1 May 2023, when the AS01 RSV vaccine became available in the USA. Individuals had to be aged 60 or older at the time of vaccination, aligning with the age range for which both vaccines are licensed. The U.S. Centres for Disease Control and Prevention recommends the recombinant shingles vaccine for anyone over 50 and the RSV vaccine for anyone over 60. Beneficiaries of Medicare have both vaccines covered under Part D of the programme. Others might have it covered by their health insurance or need to pay for it out of pocket.

Two doses of the AS01 shingles vaccine are recommended. Our analysis used the first dose as the index (to avoid potential survivorship bias that could occur if people who develop dementia after the first dose are excluded) and did not distinguish those who received one dose from those who received two (since repeated mentions of the vaccine in EHR data can be due to re-coding rather than separate doses).

A comparator cohort was established, consisting of individuals aged 60 or older who received a flu vaccine on or after 1 May 2023 (which could be adjuvanted or not, but did not contain the AS01). The choice of comparator vaccine was justified as follows. We needed to select a vaccine that can be received by older adults. This excluded vaccines that are administered once and for all in adulthood in favour of vaccines that are repeatedly administered: the flu and Tdap vaccines. In our previous study, we had compared the AS01 shingles vaccine with both the flu and Tdap vaccines^5^. Here, to reduce the number of comparisons and because both the flu and RSV infections affect the respiratory system, we focussed on the flu vaccine as a comparator.

Across all cohorts, for the primary analysis, individuals were excluded if they had any of the following diagnoses recorded before or within 3 months after vaccination:

- Vascular dementia (ICD-10-CM code F01).
- Dementia in other diseases classified elsewhere (F02).
- Unspecified dementia (F03).
- Parkinson’s disease (G20).
- Other degenerative diseases of the nervous system (G30–G32), which include all other dementias not mentioned above (e.g. Alzheimer’s disease (G30)).

The code used to identify the RSV vaccine in the EHR dataset covers both the RSVPreF3 OA vaccine Arexvy (AS01 RSV vaccine from GSK) and a smaller proportion of the RSVpreF vaccine Abrysvo (a recombinant RSV vaccine not containing AS01 from Pfizer). The brand is only specified for 44% of occurrences, with 64% of them being Arexvy (the AS01 vaccine) and 36% being Abrysvo. We excluded the latter from our cohort. Assuming that the proportion of Arexvy is the same among the vaccines recorded without a brand as it is among those with a recorded brand, we can estimate that our cohort is made up of (0.64 × 0.44 + 0.64 × 0.56)/(0.64 × 0.44 + 0.56) = 76% Arexvy.

### Covariates

Cohorts were matched for 66 covariates, including sociodemographic factors, comorbidities (capturing major body systems, and those specifically associated with dementia), history of herpes infection and history of influenza vaccination. All covariates (with ICD-10 codes for comorbidities) are listed in Supplementary Data 1. Covariates were selected as follows and follow the same strategy as in our previous study on the link between the shingles vaccine and dementia^5^.

All available sociodemographic factors were selected. These include age, sex (as recorded in the individual’s EHR), ethnicity, race, and marital status. Age is reported as mean and SD, but was matched using 2-year bins (60–61, 62–63, 64–65, etc.) up to 95 years old, and those 95 and over were grouped together. This provides tighter control on age than using age as a continuous variable.

All broad ICD-10 categories of comorbidities were then included to balance comorbidity profiles between cohorts and since indirect link with dementia can be posited for most comorbidity profiles (e.g. respiratory illness increases risk of infection and delirium and thus dementia; diseases of the ear can increase the risk of hearing loss which is a risk factor for dementia).

Some broad ICD-10 categories were further broken down into their most prevalent constituents. This includes ‘Neoplasms’ (ICD-10 codes C00-D49) that was deemed too heterogeneous (as it includes both benign and malignant neoplasms); cardiovascular diseases (I00-99) and psychiatric disorders (F10-59) given their strong link with dementia; endocrine, nutritional and metabolic disorders (E00-89) which was deemed too heterogeneous and because it contains specific risk factors for dementia such as overweight and obesity, diabetes, thyroid disorders, and vitamin B12/folate deficiency. In addition, compared to our previous study^5^, we also matched for the main autoimmune diseases affecting older adults (autoimmune thyroid diseases, rheumatoid arthritis, Sjögren syndrome, dermatopolymyositis, and Wegener’s granulomatosis). This reflects growing evidence for their association with dementia^23^. Prior herpes infections (both herpes simplex and herpes zoster) were also included.

Some factors affecting health and healthcare use (ICD-10 codes Z00-Z99) were also included based on whether they differed substantially between unmatched cohorts (SMD > 0.15) with a prevalence of at least 1 in 30 cases in either cohort.

Finally, to capture proxies of vaccine hesitancy, history of influenza vaccination (recommended every year for all adults in the USA) was included^24^.

### Outcomes

The primary outcome was a first diagnosis of dementia from 3 months (to exclude delayed diagnosis of pre-existing dementia) to 18 months post-vaccination in a time-to-event analysis. This included any of six ICD-10 codes: vascular dementia (ICD-10 code F01), dementia in other diseases classified elsewhere (F02), Unspecified dementia (F03), Alzheimer’s disease (G30), Frontotemporal dementia (G31.0), and Dementia with Lewy bodies (G31.83), as used in our previous study^5^. The follow-up extended to 25 March 2025 when the analysis was run. Individuals were followed up for 18 months or until their last contact with a healthcare organisation, whichever came first. If their follow-up was shorter than 18 months, this was accounted for in the time-to-event analysis using censoring.

Secondary outcomes included the composite of dementia or death (to assess for survivorship bias), each dementia subcategory, shingles (ICD-10 code B02) used as a positive control outcome expected to be affected by shingles vaccine and not others, as well as a composite negative control outcome of any of five acutely painful conditions not associated with dementia (acute appendicitis, acute cholecystitis, acute pancreatitis, adhesive capsulitis of shoulder, and trigeminal neuralgia) as used in our previous study^5^. The choice of a composite negative control outcome guarantees that its incidence is high enough so that the expected null associations cannot be attributed to a lack of statistical power. Associations with each individual component of the composite outcome are also reported for completeness.

### Statistical analyses

Propensity score 1:1 matching with a calliper of 0.1 was used to match cohorts on covariates. Characteristics with an SMD between cohorts <0.1 were considered well matched^25^. In propensity score matching, the propensity score was calculated using a logistic regression (implemented by the function Logistic Regression of the scikit-learn package in Python 3.7) including each of the covariates mentioned above. In order to eliminate the influence of ordering of records, the order of the records in the covariate matrix was randomised before matching. The matching itself was performed with numpy 1.21.5 in Python 3.7.

Incidences of outcomes were calculated with the Kaplan–Meier estimator. The assumption that the hazards were proportional was tested using the generalised Schoenfeld approach implemented in the cox.zph function of the survival package (version 3.2.3) in R. In doing so, the proportionality assumption was found to be violated for the primary outcome (*P* = 0.0038). Thus, the Cox proportional hazard model was not used, and the restricted mean time lost (RMTL) was used instead^26–28^. This was calculated using the R package survRM2 version 1.0.4.

The RMTL is the counterpart of the restricted mean survival time^29,30^. The ratio of RMTL has a meaningful clinical interpretation: it represents how much more time, on average, an individual has lived without the outcome during the follow-up period^26^. CIs were estimated using a parametric approach as defined in the SurvRM2 package in R^31^.

The analysis was repeated after stratifying cohorts by sex. Moderation by sex was tested using a permutation test as follows, with 1000 permutations. The RMTL ratios were first calculated independently for men and women, and their difference was recorded. In each permutation, individuals were then randomly allocated to two groups of the same size as the initial ‘women’ and ‘men’ groups, and the analysis was repeated within these groups, thus leading to the calculation of RMTL ratios. The difference in absolute value between these RMTL ratios was recorded for each permutation, generating a distribution of 1000 differences in RMTL ratios under the null hypothesis. The *P* value for the permutation test was calculated as:

$$P=\frac{1+{n}_{ > }}{1+n},$$

where *n* = 1000 is the number of permutations, and *n*_>_ is the number of permutations for which the difference in RMTL ratios was greater (in absolute value) than that observed in the non-permuted dataset.

The analysis was also repeated after expanding the follow-up window to include the first 3 months post-vaccination, and including those who developed dementia during that time window.

Significance for all tests was set at two-sided *P* < 0.05. Analyses were conducted in R 4.2.1.

## Supplementary information

Supplementary Information

Supplementary Data 1 and 2

## Data Availability

The TriNetX system returned the results of these analyses as CSV files, which we downloaded and archived. Aggregate data, as presented in this article, can be freely accessed by anyone at https://osf.io/jkpqw. The data used for this article were acquired from TriNetX. This study had no special privileges. Inclusion criteria specified in the Methods would allow other researchers to identify similar cohorts of patients as we used here for these analyses; however, TriNetX is a live platform with new data being added daily, so exact counts will vary. To gain access to the data, a request can be made to TriNetX (join@trinetx.com), but costs might be incurred, and a data sharing agreement would be necessary.

## Code availability

The code used to generate the results of this analysis can be freely accessed at https://osf.io/jkpqw.
