# Supplementary material for: Lower risk of dementia with AS01-adjuvanted vaccination against shingles and respiratory syncytial virus infections
Source: NPJ Vaccines. 2025 Jun 25;10:130. doi: 10.1038/s41541-025-01172-3 (PMC12198376; doi:10.1038/s41541-025-01172-3)
Supplement: Supplementary file 1 — Supplementary Information [file 41541_2025_1172_MOESM1_ESM.pdf]

Supplementary information

Supplementary Data Titles

Supplementary Data 1 All baseline characteristics compared between cohorts after matching for the analysis of dementia risk

Supplementary Data 2 Results for secondary outcomes within 1 year of vaccination
